# SCA2 presenting as a focal dystonia

**DOI:** 10.1186/s40734-018-0073-7

**Published:** 2018-08-13

**Authors:** Nan Cheng, Heather M. Wied, James J. Gaul, Lauren E. Doyle, Stephen G. Reich

**Affiliations:** 1 Department of Neurology, University of Maryland School of Medicine, Baltimore, MD USA; 2 Department of Pediatrics, Cincinnati Children’s Hospital Medical Center, Cincinnati, OH USA; 3 Bucks Neurological Group, Langhorne, PA USA; 4 Department of Genetic Counseling, University of North Carolina Greensboro School of Health and Human Sciences, Greensboro, NC USA

**Keywords:** Spinocerebellar Ataxia 2, SCA2, Dystonia, Deep brain stimulation

## Abstract

**Background:**

Spinocerebellar ataxia 2 (SCA2) is an autosomal dominant neurodegenerative disorder caused by CAG repeat expansions in *ATXN2* on chromosome 12q24. Patients present with adult-onset progressive gait ataxia, slow saccades, nystagmus, dysarthria and peripheral neuropathy. Dystonia is known to occur as SCA2 advances, but is rarely the presenting symptom.

**Case presentation:**

A 43-year-old right handed woman presented with focal dystonia of the right hand which started two years earlier with difficulty writing. There were only mild cerebellar signs. Her mother was reported to have a progressive gait disorder and we subsequently learned that she had SCA2. A total of 10 maternal family members were similarly affected. Over the course of 10 years, the patient’s cerebellar signs progressed only mildly however the dystonia worsened to the extent of inability to use her right hand. Dystonia did not improve significantly with botulinum toxin, levodopa or trihexyphenidyl, but has shown marked improvement since DBS implantation in the GPi.

**Conclusions:**

We describe a patient with SCA2 who presented with focal dystonia of the right upper extremity. Subtle cerebellar signs as well as the family history became especially important given the absence of predominant gait ataxia. Our case emphasizes that focal dystonia is not only a feature of SCA2, but can also rarely be the presenting sign as well as the most prominent feature during the disease course.

**Electronic supplementary material:**

The online version of this article (10.1186/s40734-018-0073-7) contains supplementary material, which is available to authorized users.

## Background

The spinocerebellar ataxias (SCAs) are a group of inherited neurodegenerative disorders that encompass a broad range of neurologic signs. SCAs are characterized prominently by cerebellar ataxia along with variable oculomotor abnormalities, pyramidal and extrapyramidal features (EPS), dementia and peripheral neuropathy [1]. There are over 44 distinct subtypes of SCAs [2], with the more prevalent types – SCA1, SCA2, SCA3 and SCA6 – caused by autosomal dominant CAG repeat expansions in polyglutamine encoding genes [3].

SCA2 is caused by repeat expansions in the ataxin-2 gene on chromosome 12q24. Neuropathologically, SCA2 is characterized by cerebellar and brainstem atrophy, as well as basal ganglia, thalamic, spinal cord and peripheral nerve degeneration [4] [5]. As with all the SCAs, the clinical hallmark and presenting sign is a progressive cerebellar syndrome. Associated clinical features include slow saccades, muscle cramps and peripheral neuropathy. Less commonly, patients can present with extra-pyramidal signs (EPS) such as levodopa responsive or atypical parkinsonism [6], REM sleep behavior disorder and dystonia [7]; however these are rarely the initial sign. The diagnosis of an SCA is confirmed by genetic testing, but if testing is not possible or consented then physical findings and a family history become paramount. Given the emphasis on ataxia as a hallmark symptom, when SCA2 begins as dystonia with minimal ataxia, the correct diagnosis may not be considered.

## Case presentation

A 43-year-old right handed woman presented in 2006 with difficulty writing, impaired dexterity of the right hand, and mild unsteadiness with walking. Her sole initial symptom was difficulty writing with the right hand and with time, other dexterous movements with the right hand became affected. Her left hand was asymptomatic. On examination, she scored 30 on the MMSE. There was a subtle dysarthria. She walked well except for a slight tendency to veer to either side. There was mild bilateral upper extremity dysmetria and mild ataxia with heel-to-shin. With the hands outstretched, as well as when performing movements with the right hand (especially writing), the proximal and distal thumb flexed involuntarily into the palm. When attempting to write, there was flexion of the right thumb, extension of the wrist, and excessive flexion at the PIP joint of all fingers. She did not notice any sensory tricks for dystonia and did not have mirror dystonia. Her reflexes were 2+ in the upper extremities and 3+ in the lower extremities. Brain MRI demonstrated cerebellar and brainstem atrophy.

**Fig. 1:**
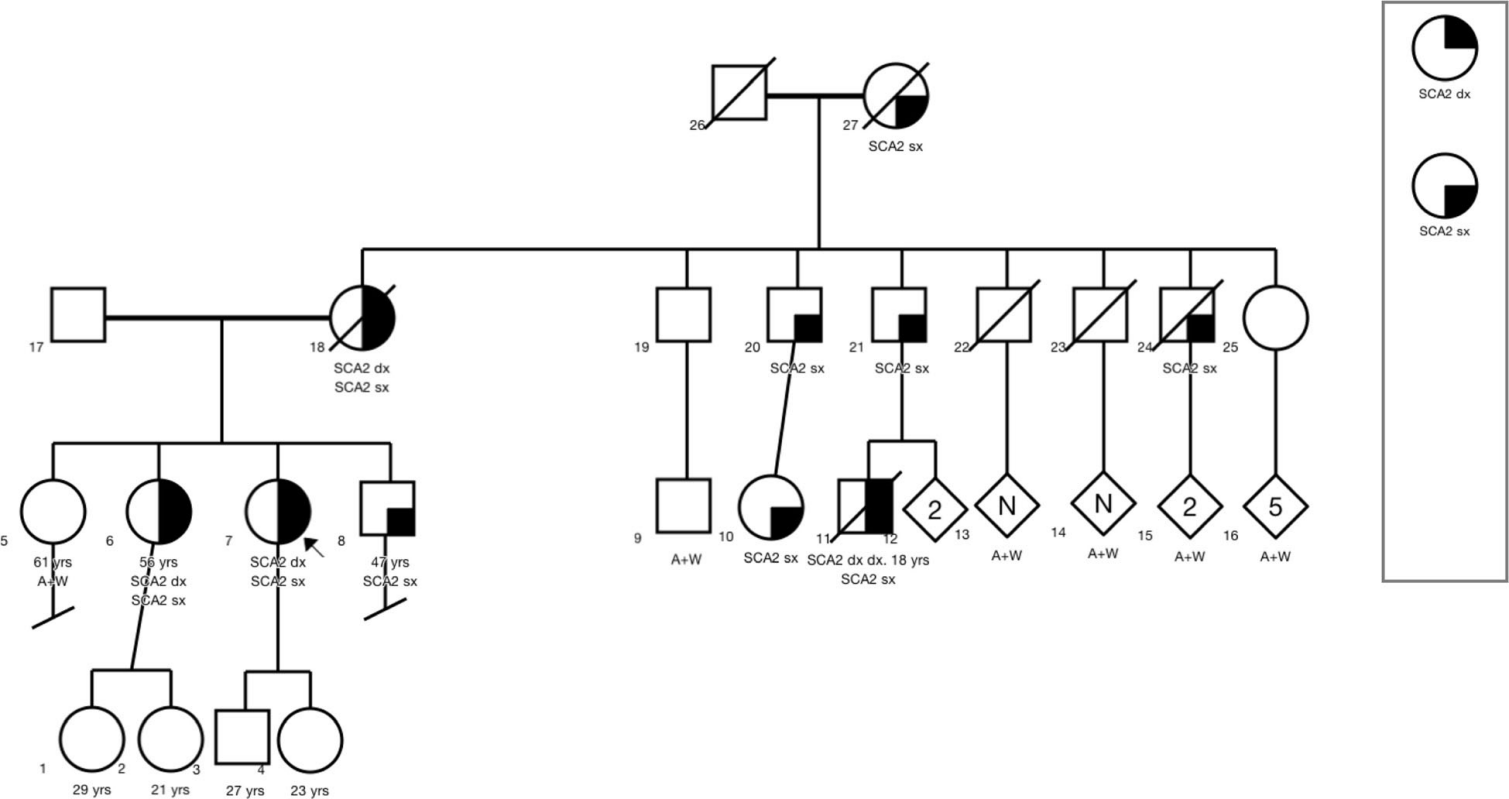
Three generation pedigree with a total of 10 affected family members. Colored shapes indicate affected individuals based on testing, history or both. Patient is indicated by arrow

Her mother was reported to have a progressive gait disorder, presenting with unsteadiness, hand clumsiness and dysarthria. We later learned that her mother had been diagnosed with SCA2 with 37 CAG repeats. Prior to death, her mother had a resting tremor, nystagmus, and cervical dystonia. Our patient’s maternal grandmother, two of three siblings and several maternal uncles and cousins were all similarly affected; a total of ten individuals were considered symptomatic in a three generation pedigree (Fig. 1).

The patient was not seen again until eight years later, at age 51. During that time, there had been only mild progression of cerebellar symptoms but the dystonia of the right hand continued to worsen to the extent that she was barely able to write and the dystonia also impaired many other tasks with the right hand. On examination, dysarthria remained mild. There was severe writer’s dystonia (Additional file 1: Video 1) with excessive flexion of the thumb and all digits, poor control of the pen, and extension of the wrist. With the hands outstretched there was flexion of the right thumb into the palm and mild extension of the first finger at the MCP joint. There was moderate dysmetria with finger-to-nose on the right. There was slight dysdiadokokinesia of the left hand and mild dysmetria with finger-to-nose on the left. There was mild gait ataxia. Although the saccades were not overtly slow, when making saccades between horizontal targets, they were almost always performed with a simultaneous blink, which can be sign of subtle slowing or difficulty in initiation [8]. There was no significant improvement with 600 mg per day of levodopa, the maximally tolerated dose of trihexyphenidyl, as well as botulinum toxin. As such, in 2015, at age 52, she underwent deep brain stimulation (DBS) of the left globus pallidus internus (GPi), resulting in significant improvement in the dystonia. The monopolar Medtronic device achieved optimal settings at: Lead C + 0-, frequency of 165 Hz, voltage of 2.4 V and PW of 450 μs.


**Additional file 1:** Video 1. This video was performed in 2014, at age 51. There is severe writer’s dystonia with excessive flexion of the thumb and all digits, poor control of the pen, and extension of the wrist. With the hands outstretched there is flexion of the right thumb into the palm, flexion of the distal thumb, and mild extension of the first finger at the MCP joint. The dystonia of the right hand, especially flexion of the thumb, worsens when opening and closing the right hand. There is moderate dysmetria with finger-to-nose on the right. There is slight dysdiadokokinesia of the left hand and mild dysmetria with finger-to-nose on the left. Although the saccades are not overtly slow, when making saccades between horizontal targets, they are almost always performed with a simultaneous blink. There is mild gait ataxia with a tendency to veer. (MP4 155136 kb)


When seen 2 years after DBS (Additional file 2: Video 2), there was much less spontaneous flexion of the right thumb into the palm with the arms outstretched. Although not shown on the video, there was marked improvement in her ability to write within the first several months following DBS. Compared to pre-operatively, she performed finger-to-nose better on the right suggesting that dystonia rather than ataxia was the main cause of impairment prior to DBS. The dysmetria of the left upper extremity had progressed but there was still only mild dysdiadokokinesia bilaterally. The gait ataxia had progressed but she was still ambulatory without an assist device and reported no falls. She was unable to tandem walk.


**Additional file 2:** Video 2. This video was taken 2 years after DBS. There is much less spontaneous flexion of the right thumb into the palm with the arms outstretched. Although not shown on the video, there was marked improvement in her ability to write. Compared to pre-operatively, she performs finger-to-nose better on the right suggesting that dystonia rather than ataxia was the main cause of impairment prior to DBS. The dysmetria of the left upper extremity has progressed but there is still only mild dysdiadokokinesia bilaterally. The gait ataxia has progressed but she is still ambulatory without an assist device. She is unable to tandem walk. (MP4 99104 kb)


## Discussion

A variety of extrapyramidal signs have been observed in the autosomal dominant ataxias. As many as 60% of SCA2 patients experience EPS, most frequently in the form of REM behavior disorder (48.48%), parkinsonism (27.27%), dystonia (27.27%) and postural tremor (20.9%) [1]. Boesch et al. observed cervical dystonia (CD) as the only dystonic sign in 61% of their 18 SCA2 patients but it was the initial symptom in only one [9]. Walsh et al. reported on 2 siblings with SCA2 where CD preceded gait ataxia by decades, and for one of the siblings CD was the presenting sign [10]. However, the spectrum of dystonia in SCA2 has been expanded in more recent studies. Jhunjhunwala et al. observed dystonic signs in 17.9% of patients in the forms of generalized, cervical, facial and lingual and foot dystonia [1].

Dystonia has been observed in other SCAs including SCA1, SCA6, SCA7 and SCA14 [11]. In SCA1, one case of writer’s cramp manifested 1.5 years after gait ataxia but became the primary disabling complaint [12]. In another case, writer’s cramp was the presenting sign as well as the most prominent disease feature [13]. In SCA6, a case of writer’s cramp predated ataxia by 5 years [14] and manifested in 3/5 family members in a second report [15]. Similarly in SCA14, task specific writer’s cramp predated ataxia by 10 years [16] [17] and was observed in four other German families [17].

Several studies have attempted to correlate dystonic signs with genetic markers and prognostic value [18]. Normal SCA2 alleles have 14–31 repeats [19] [20], with most wild type alleles showing 22 repeats. The pathologic range of CAG repeats is from 34 to 200. Cancel et al. observed dystonia in 9/104 patients with SCA2 and postulated that dystonia presented in patients with larger CAG repeats [19]. However, a recent study by Kuo et al. examining dystonia in 334 patients with SCA1, 2, 3 and 6 patients only observed this correlation between dystonia, earlier age of onset (10 years) and longer repeat length in SCA3 [21]. Although patients with SCA1, 2, or 3 and dystonia had higher SARA scores indicating higher impairment, the presence of dystonia had no influence on the rate of progression; in contrast to SCA6, dystonia was associated with a slower rate of progression. The authors hypothesized that dystonia in the SCAs could result from as yet determined gene-gene interactions in which the repeat expansions produced protein products that preferentially affect different pathways and result in varying disease progression and phenotypes.

The pathophysiology of dystonia in SCA2 is unclear. SCA2 pathology involves the basal ganglia, thalamocortical and cerebello-thalamo-cortical circuits [5] [22] [23] [24]. A study of 18F-dopa metabolism confirmed nigrostriatal dopaminergic involvement in SCA2, with the caudate severely affected [25]. When comparing SCA1 and SCA3, Durr et al. found significantly more GPi and STN degeneration in SCA3, which may account for the predominance of dystonia in SCA3 compared to its relative rarity in SCA1 [20]. Since SCA2 shows the same neuropathology as SCA3, basal ganglia involvement may explain the increased incidence of dystonia in both SCA2 and SCA3 versus SCA1.

In addition to the basal ganglia, cerebellar dysfunction has also been implicated in dystonia [26]. Cerebellar atrophy in SCA2 is evident as early as seven years before symptom onset [27]. Secondary CD was most commonly associated with structural cerebellar and brainstem lesions and less commonly with basal ganglia pathology [28] [29]. In addition, a case of secondary focal limb dystonia was reported in a patient with a primary cerebellar mass [30]. Autopsy studies of patients with CD have shown degeneration in cerebellar Purkinje cells [31]. A voxel based morphometry and diffusion weighted MRI study on task specific writer’s cramp found pathological changes in the cerebellum, as well as other anatomic areas responsible for sensorimotor control during writing and speaking [32]. Specific to focal hand dystonia, transcranial direct current stimulation (tDCS) of the cerebellum has shown to improve handwriting [33] [34]. When studying DBS to the ventral intermediate nucleus (Vim) in SCA patients, Hashimoto et al. proposed that DBS disrupted cerebellar sensory feedback, thereby reducing motor modulation of the cerebello-thalamo-cortical network [35].

DBS has shown success in improving tremor and dystonia in SCAs [36] [37], but at the cost of worsening ataxia. In one SCA2 case, DBS resolved the tremor, but the patient sustained worsening ataxia to the extent of requiring inpatient rehabilitation for two months post operation [38]. In a SCA17 case, DBS resolved the dystonic tremor, but the patient experienced new onset gait and appendicular ataxia and required the use of a power scooter [38]. However a recent study of five SCA patients with Vim DBS reported no improvement or worsening of ataxia [35].

As is the case with other neurodegenerative diseases, such as Parkinson’s disease, Huntington’s disease and Alzheimer’s, there is great interest in identifying the earliest features of the SCAs, even prior to the onset of ataxia [39]. This is known as the prodromal stage [40]. Globas et al. found that one-third of patients with SCA 1, 2, 3 and 6 presented with a complaint other than ataxia [41]. In SCA2, prodromal symptoms include muscle cramps, sensory abnormalities, hyperreflexia, olfactory dysfunction, dysautonomia, cognitive decline, and impaired handwriting [40] [42]. Our patient potentially expands the prodromal stage by demonstrating that focal dystonia may precede ataxia in SCA2.

## Conclusion

Discovery of the genes for SCAs has greatly increased the diversity of the phenotype, which paradoxically may not include ataxia as a presenting sign. This led Gwinn-Hardy to pose the riddle: “When is Ataxia Not Ataxia?” [6] The answer is: when it is dystonia or parkinsonism. Our case emphasizes that focal dystonia is not only a feature of SCA2, but may be the presenting sign as well as the most prominent feature during the disease course. In addition, DBS provided significant improvement for dystonia without worsening ataxia.
